# Effect of pH on the solubility and volumetric change of ready-to-use Bio-C Repair bioceramic material

**DOI:** 10.1590/1807-3107bor-2024.vol38.0028

**Published:** 2024-04-05

**Authors:** Luana Raphael da SILVA, Jader Camilo PINTO, Juliane Maria GUERREIRO-TANOMARU, Mário TANOMARU-FILHO

**Affiliations:** (a) Universidade Estadual Paulista – Unesp, Araraquara School of Dentistry, Department of Restorative Dentistry, Araraquara, SP, Brazil.

**Keywords:** Endodontics, Dental Materials, X-Ray Microtomography

## Abstract

Acidic pH can modify the properties of repair cements. In this study, volumetric change and solubility of the ready-to-use bioceramic repair cement Bio-C Repair (BCR, Angelus, Londrina, PR, Brazil) were evaluated after immersion in phosphate-buffered saline (PBS) (pH 7.0) or butyric acid (pH 4.5). Solubility was determined by the difference in initial and final mass using polyethylene tubes measuring 4 mm high and 6.70 mm in internal diameter that were filled with BCR and immersed in 7.5 mL of PBS or butyric acid for 7 days. The volumetric change was established by using bovine dentin tubes measuring 4 mm long with an internal diameter of 1.5 mm. The dentin tubes were filled with BCR at 37°C for 24 hours. Scanning was performed with micro-computed tomography (micro-CT; SkyScan 1176, Bruker, Kontich, Belgium) with a voxel size of 8.74 µm. Then, the specimens were immersed in 1.5 mL of PBS or butyric acid at and 37 °C for 7 days. After this period, a new micro-CT scan was performed. Bio-C Repair showed greater mass loss after immersion in butyric acid when compared with immersion in PBS (p<0.05). Bio-C Repair showed volumetric loss after immersion in butyric acid and increase in volume after immersion in PBS (p<0.05). The acidic pH influenced the solubility and dimensional stability of the Bio-C Repair bioceramic cement, promoting a higher percentage of solubility and decrease in volumetric values.

## Introduction

Bioceramic materials must have proper physicochemical properties.^1^ Bio-C Repair (BCR, Angelus, Londrina, Brazil), a ready-to-use bioceramic repair cement, is composed of tricalcium silicate, calcium aluminate, calcium oxide, zirconium oxide, iron oxide, silicon dioxide and a dispersing agent. BCR has similar cytocompatibility and a higher radiopacity than Biodentine (Septodont, Saint Maur des Fosses, France).^2,3^ Bio-C Repair is not cytotoxic to osteoblastic cells,^4^ and promoted the deposition of mineralized nodules and cell migration^5^ demonstrating its biocompatibility and bioactive potential.^6^ BCR showed low volumetric change after immersion in distilled water, with values lower than 3%, after evaluation by micro computed tomography (micro-CT).^7^

Dimensional stability and low solubility are essential properties required for endodontic materials, as they reduce the possibility of bacterial infiltration.^8^ According to the guidelines of both the International Organization for Standardization (ISO),^9^ and American Dental Association (ANSI/ADA),^10^ the solubility of endodontic cements must not exceed 3.0% of mass loss and the change in Dimension (size) should not exceed values of 1.0% shrinkage or 0.1% expansion.^11^ The ISO 6876/2012 solubility test^6^ has limitations for the purpose of evaluating calcium silicate-based materials due to the hydrophilic nature of bioceramic cements.^12^ As regards dimensional changes, single-direction assessment is limited to demonstrating changes in contraction or expansion.^13,14^

The physicochemical properties of repair materials can be changed in an acid medium.^15^ An acidic pH may occur in the periapical region with the presence of an inflammatory reaction,^16^ leading to changes in the dimensional stability of bioceramic repair materials^15,17^and promoting volumetric loss.^15^ However, materials can behave differently in an acidic environment. Acidic pH improved the sealing ability of Geristore and MTA with CPC matrix.^18^ Methodologies that simulate clinical conditions such as acidic pH may allow a better understanding of the physicochemical properties of calcium silicate-based cements.^19^ Butyric acid is used to simulate the clinical conditions of acidic pH,^15,17^ since this material is a by-product of anaerobic bacterial metabolism.^20^

Immersion of bioceramic cements in simulated body fluids such as phosphate-buffered saline (PBS) allows clinical conditions to be simulated more closely and has demonstrated lower solubility for calcium silicate-based materials when compared with immersion in distilled water.^20^ Lower solubility values after immersion in PBS may be related to the interaction of calcium ions arising from bioceramic cements and phosphate from the simulated body fluid that allow the formation of a surface layer of hydroxyapatite in calcium silicate-based materials.^8^

Several methods have been proposed for evaluating the properties of endodontic materials. Micro-CT is a non-destructive three-dimensional analysis method capable of complementing conventional ISO/ADA tests.^9,10,21^ This methodology can be used to evaluate the volumetric behavior of endodontic cements after immersion in different media.^22,23^ However, to date, there have been no studies that have evaluated the effect of the immersion medium pH on the solubility and volumetric change of BCR. Thus, the aim of this study was to evaluate the effect of immersion in PBS (pH 7.0) or butyric acid (pH 4.5) on the solubility and volumetric change of the ready-to-use bioceramic cement, BCR, by means of micro-CT. The null hypothesis was that the pH of the different immersion media would not influence the solubility and volumetric change of BCR cement.

## Methodology

### Sample size calculation

The tests were performed with a specific software G * Power 3.1.7 for Windows (Heinrich-Heine-Universitat Dusseldorf, Dusseldorf, Germany). The two independent means t-test was used with an Alpha error of 0.05 and a Beta power of 0.95. Previous studies were used to determine the specific effect size for volumetric change, 2,28,^24^ and solubility, 2,37.^8^ Since the effect size was similar for both variables, a total of 7 specimens per group was indicated as the ideal sample size required for volumetric change and solubility.

### Specimen preparation and scanning by micro-CT

This study was approved by the Committee on Ethics in the Use of Animals (CEUA: No. 37/2020). Extracted bovine teeth were selected from images on digital radiographs (Kodak RVG 6100 Digital Radiography System, Marne-la-Vallée, France) to confirm the absence of anomalies. The roots were cross-sectioned with a carborundum disc (Dentorium Products Co. Inc., Farmingdale, New York), to obtain 4 mm long specimens. Subsequently, a single previously trained and calibrated operator prepared the root canals using Gates-Glidden drills number 6 (Dentsply Maillefer, Ballaigues, Switzerland) to manufacture cylindrical tubes with an internal diameter of 1.5 mm and wall thickness of approximately 1 mm During preparation, the root canals were inundated with 1% sodium hypochlorite (NaOCl) solution. Final irrigation was performed with 5 mL of 2.5% NaOCl and 5 mL of 17% EDTA, for 3 minutes, followed by distilled water. After preparation, the specimens were immersed in distilled and deionized water and stored in an oven at 37°C for 24 hours. After 24 hours had elapsed, the cavities were filled with Bio-C Repair (n = 8). Due to the consistency of the repair material, a condenser kit (Ref.: 324501, Nos. 2, 3 and 4; Golgran; São Caetano do Sul, Brazil) was used to perform the filling. The samples were kept in an oven at 37°C humidity 95% for 24 hours. After this period, the specimens were submitted to initial scanning by micro-CT (SkyScan 1176, Bruker, Kontich, Belgium), using the following parameters: copper and aluminum filter, frame 4, rotation step of 0.5, rotation of 180º, 80 kV, 300 mA and voxel size of 8.74 µm.

After the initial scanning of the cavities filled with the material, the specimens were immersed in 1.5 ml of PBS (pH 7.0) or butyric acid (Sigma; pH 4.5) and kept in an oven at 37ºC for 7 days. The butyric acid solution was changed every 24 hours. New micro-CT scans were performed after 7 days using the same parameters as those previously described.

### Volumetric change

**Figure 1:**
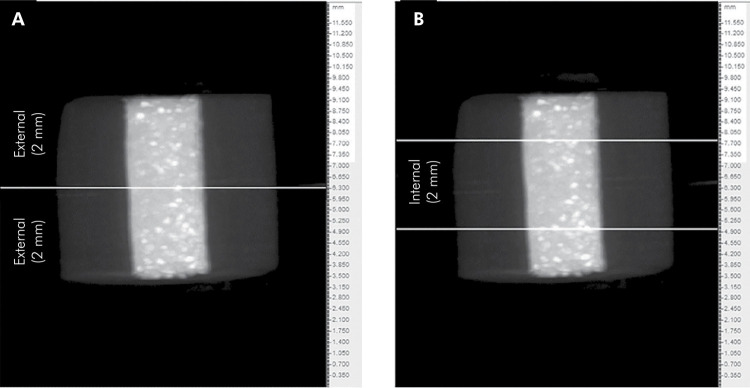
Representative image of the sample division into 3 parts for volumetric change analysis using the CTAn software. A, extremity and B, internal portion.

The images were reconstructed with use of the NRecon program (V1.6.4.7; SkyScan, Belgium). Geographic alignment of the images in the different experimental periods was performed using the “3D registration” function of the Data Viewer software (V1.5.2.4; SkyScan, Belgium). Quantitative analyses of the images were performed using the CTAn software (V1.15.4.0; SkyScan, Belgium). For analyzing the volumetric change, each specimen was divided into three parts: 2 mm for each extremity and 2 mm in the center of the sample (internal portion), as shown in Figure 1. For this purpose, the cross section representing the middle portion of the specimen was determined in the CTAn software, and from this point on, the entire top extension of the sample (extremity) was added to the region of interest for volumetric analysis. The same procedure was repeated for the bottom portion (extremity). For analysis of the internal portion, 1 mm of the top and 1 mm of the bottom portions were removed from the region of interest, thus only the central 2 mm were considered. In this way, the difference in the total volume of the materials, in mm^3^, was calculated before and after the immersions. The grayscale range needed for recognizing each study object was determined in a density histogram using adaptive thresholding. Three-dimensional models were created by using the CTVox software (v.3.2, Bruker-microCT).

### Solubility

The solubility test was performed using a modified methodology derived from ISO 6876 and ANSI/ADA No. 57. Polyethylene tubes 4 mm high with an internal diameter of 6.70 mm (n=7) were filled with BCR using a condenser kit (Ref.: 324501, Nos. 2, 3 and 4; Golgran; São Caetano do Sul, SP, Brazil). After filling, the samples were covered with gauze that had been wet with distilled and deionized water and were then kept in an oven at 37ºC for 24 hours. After this, the polyethylene tubes were removed, the samples were abraded with abrasive paper to obtain smooth and uniform surfaces and left in the desiccator for 24 hours. After 24 hours had elapsed, the specimens were weighed on an HM-200 precision balance (A&D Engineering, Inc., Bradford, MA, USA) daily until stabilization of the initial mass occurred (difference of up to 0.0002). Afterwards the samples were immersed in plastic receptacles containing 7.5 mL of PBS (pH 7.0) or butyric acid (Sigma; pH 4.5) and kept in an oven at 37ºC for 7 days. The butyric acid solution was changed every 24 hours. After 7 days the samples were placed in a desiccator again for 24 hours. After this, the samples were weighed daily until the final mass was stabilized. Differences between initial (IM) and final (FM) mass were recorded with the value close to 0.0001 g. Differences in weight were calculated in % and had to be less than 3% per in accordance with the ISO and ANSI/ADA standards. The percentage of solubility was calculated in the following manner:


$$(IM-FM)/{IM}^{*}100$$

### Statistical analysis

All data were submitted to the Shapiro Wilk normality test, and Volumetric Change values were found to be not normally distributed, however, solubility values were shown to be normally distributed. The Mann Whitney test for volumetric change and the unpaired t test for solubility were used for comparisons between groups with a significance level of 5%.

## Results

### Solubility

**Figure 2:**
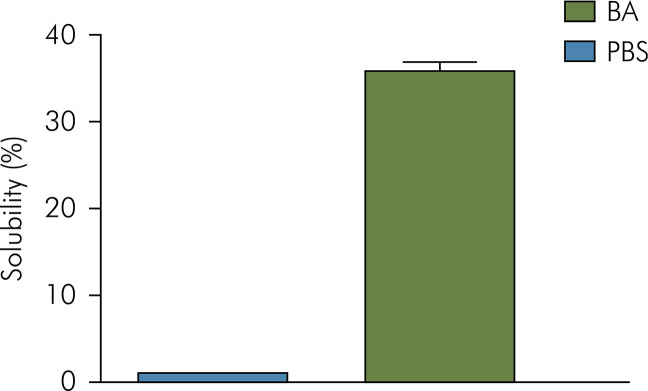
Bar graph showing mean and standard deviation of solubility (%) observed for BCR cement after 7 days of immersion in PBS or butyric acid.

The immersion medium used significantly affected the solubility of Bio-C Repair (p < 0.05). When immersed in butyric acid, Bio-C Repair showed an average solubility of 35.76%. The opposite behavior was observed when this material was immersed in PBS, when it showed a mean gain in mass of 0.65% (Figure 2).

### Volumetric change

**Figure 3:**
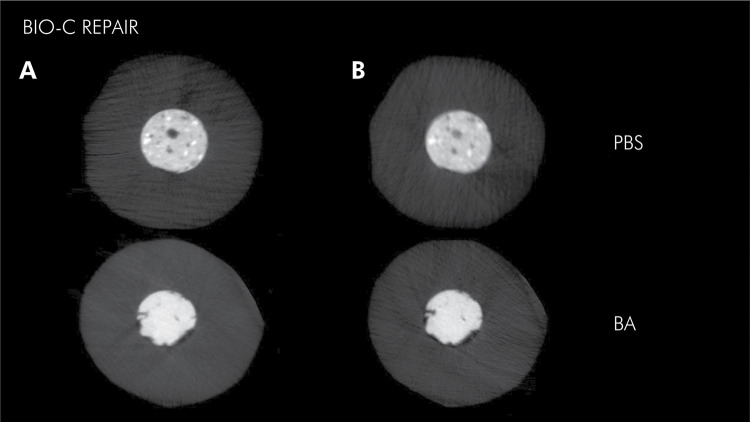
Cross-sections of micro-CT images, obtained by using the CTAn software, showing the Bio-C Repair cement before (A) and after (B) immersion in PBS or butyric acid for 7 days.

Bio-C Repair showed volumetric loss after immersion in butyric acid, while immersion of the material in PBS led to a gain in volume (p < 0.05). The regions analyzed (extremities and inner portion) showed similar volumetric behavior irrespective of the immersion medium used (p > 0.05). (Table and Figure 3).

**Table:** Median, minimum and maximum of the percentage of volumetric change of the extremity and internal portion of the Bio-C Repair cement tested in different immersion media.

Variable	PBS	Butyric acid
Extremity	0,5760 (0,1112-2,2529)^a^	-0.5837[-2.8335–( -0.2402) ^] b^
Internal portion	0.3590 (-1.785 -2.0027) ^a^	-0.4709[-1.4254–( -0.0320) ^] b^

Different superscript lower case letters in the same line indicate statistical difference between the groups (p < 0.05) There was no significant difference between the extremity and internal portion for the same medium (p > 0.05).

## Discussion

In the present study, the effect of pH on the solubility and volumetric change of BCR repair cement was evaluated. Bovine dentin tubes, which more closely represent the clinical conditions and interaction of the bioceramic material with dentin, were used.^25^ The null hypothesis was rejected since BCR showed higher solubility and volumetric change values after immersion in the butyric acid solution. Other powder-liquid bioceramic repair cements have shown volumetric loss after immersion in butyric acid.^15,26^ A persistent infection in the periapical region can result in an acidic environment generated by bacterial by-products.^20^ The acidic environment is capable of interfering in the formation of hydroxyapatite crystals, altering the hydration process of bioceramics, and increasing the dissolution of materials, thereby promoting volume loss.^15,26^ Furthermore, acidic pH can impair surface hardness,^17^ compressive strength,^27^ and lead to increasing degree of porosity of repair materials.^17^

Whereas the Bio-C Repair immersed in acid presented a high solubility value and micro-CT analysis revealed a volumetric loss lower than 1%. Although the solubility and volumetric stability are directly related, calcium silicate-based cements are hydrophilic materials which, in contact with water, can disintegrate or absorb water causing dimensional changes.^11^ Micro-CT is a non-destructive three-dimensional analysis method that evaluates the volumetric behavior of endodontic materials after immersion in different solutions.^20,22^ Although micro-CT is a standardized and reproducible analysis method, it is important to note that the mass loss of the conventional solubility method may not be directly related to the volumetric changes evaluated by micro-CT.^14^ Therefore, the use of Micro-CT should be considered a valuable complement to the tests recommended by ISO and ANSI/ADA standards.

In this study, the volumetric changes observed after immersion of BCR in butyric acid or PBS could be considered low, since they remained below 3% (median below 1%), suggesting adequate volumetric behavior. A previous study demonstrated low volumetric change for BCR after immersion in distilled water.^7^ An increase in volume has been demonstrated for bioceramic repair materials presented in the powder-liquid form after immersion in PBS.^15^ In addition, micro-CT analyses of dentin tubes filled with BCR implanted into subcutaneous tissues of rats showed a low porosity and interface voids.^6^

After immersion in PBS, BCR showed negative solubility values that represented a gain in mass. Torres et al.^23^ also pointed out a gain in mass for BIO after immersion in PBS and in the same way as shown in our study, also demonstrated that this material remained above the minimum level recommended by ISO 6876. This was because in hydraulic cements, the solubility was compensated by the absorption of fluids.^13^ This result may have been related to the combination of calcium ions from bioceramic cements and phosphate from the immersion solution, leading to formation of carbonated apatite,^28^ which could result in gain in volume. Furthermore, the interaction of bioceramic materials in contact with dentin promotes precipitation of a layer of hydroxyapatite at the interface with the dentin structure.^29^ Based on the findings of the present study, the different conditions of pH affected the volumetric behavior of the Bio-C Repair bioceramic cement. However, although there are no specific norms regarding evaluations performed by micro-CT, Bio-C Repair showed volumetric stability and a potential for clinical application even in an environment with acidic pH.

## Conclusions

In conclusion, the immersion media influenced the solubility and volumetric change of the ready-to-use Bio-C Repair material that had a higher percentage of solubility after immersion in butyric acid when compared with PBS, whereas it showed volumetric loss in butyric acid and gain in volume in PBS. Thus, the results obtained in the present study could serve as a reference for future investigations using ready-to-use bioceramic repair materials.
